# Repair of human periodontal bone defects by autologous grafting stem cells derived from inflammatory dental pulp tissues

**DOI:** 10.1186/s13287-016-0404-2

**Published:** 2016-09-22

**Authors:** Ye Li, Shanmei Zhao, Xi Nan, Hong Wei, Jianfeng Shi, Ang Li, Jianzhong Gou

**Affiliations:** 1 Key Laboratory of Shaanxi Province for Craniofacial Precision Medicine Research, College of Stomatology, Xi’an Jiaotong University, Xi’an, Shaanxi China; 2 Department of Periodontology, College of Stomatology, Xi’an Jiaotong University, Xi Wu Road No. 98, Xi’an, Shaanxi 710004 China

**Keywords:** Dental pulp stem cell, Inflamed pulp, Tissue regeneration

## Abstract

**Background:**

Recently, stem cells derived from inflammatory dental pulp tissues (DPSCs-IPs) have demonstrated regenerative potential, but the real effect remains to be examined. This pilot study attempted to isolate DPSCs-IPs from two patients and to evaluate the feasibility and the effect of reconstructing periodontal intrabone defects in each patient.

**Methods:**

DPSCs-IPs were harvested from two patients with periodontal intrabone defects with their approval. After discussing the biological characteristics of DPSCs-IPs in each patient, DPSCs-IPs were loaded onto the scaffold material β-tricalcium phosphate and engrafted into the periodontal defect area in the root furcation. After 1, 3, and 9 months, the outcome was evaluated by clinical assessment and radiological study. Furthermore, new samples were collected and the biological characteristics of DPSCs-IPs were further studied compared with normal dental pulp stem cells. The primary cell culture success rate, cell viability, cell cycle analysis, and proliferation index were used to describe the growth state of DPSCs-IPs. In-vitro differentiation ability detection was used to further discuss the stem cell characteristics of DPSCs-IPs.

**Results:**

As expected, DPSCs-IPs were able to engraft and had an effect of regeneration of new bones to repair periodontal defects 9 months after surgical reconstruction. Although the success rate of primary cell culture and growth status was slightly inhibited, DPSCs-IPs expressed comparable levels of stem cell markers as well as retaining their multidifferentiation ability.

**Conclusions:**

We developed a standard procedure that is potentially safe and technological for clinical periodontal treatment using human autologous DPSCs-IPs.

**Trial registration:**

According to the editorial policies, the present study is a purely observational study, so trial registration is not required.

## Background

Periodontitis is a kind of chronic disease prevalent worldwide, featured by a loss of support tissues around the teeth, resulting in damage which continues until the teeth fall out [1]. The final goal of treatment for periodontitis is to repair the lost periodontal support tissues, especially the bone. In recent years, the rapid development of tissue engineering has shown great potential for applications in reconstruction of periodontal-associated bone defects [2–6]. In particular, the discovery of dental pulp stem cells (DPSCs) and other odontogenic stem cells has furnished new prospects for the repair of periodontal tissue [7, 8]. However, a limitation for clinical application may be the availability of autologous DPSCs, particularly for patients who have already had dental pulp disease and are not willing to sacrifice normal dental pulp tissues. Moreover, medical wastes often occur and are discarded when inflammatory pulp tissues are removed by pulpectomy.

Recently some studies found that a certain proportion of ectomesenchymal stem cells were contained within the inflammatory dental pulp tissues with retained potential for tissue regeneration [9–11]. If such tissues could be used as a kind of available resource in periodontal tissue regeneration, this may provide a way of making use of the discarded tissue as well as enabling treatment of periodontal bone defects without damaging the normal dental pulps.

However, previous studies concentrated only on the biological characteristics of stem cells isolated from inflammatory dental pulp tissues (DPSCs-IPs), without providing enough information on whether this kind of stem cell can be used in the clinical process and to determine the effectiveness of regeneration. To address these issues, the current study utilized DPSCs-IPs in periodontal treatment with the patient’s consent to provide primary evidence for future clinical application and to provide more details of DPSCs-IPs compared with two kinds of normal DPSCs.

## Methods

### Patient enrollment

Two female patients diagnosed with combined periodontal–endodontic lesions with pocket depth from 5 to 6 mm were chosen. Patient No. 1 is 30 years old with 29 teeth; Patient No. 2, aged 38, has 30 teeth left. Patients were first informed to consent to the entire treatment. The selected patients should be in accordance with the following inclusion criteria: age range from 18 to 40 years without systemic disease, no pregnancy or smoking, and no use of recreational drugs. Patients were excluded if they had undertaken any initial treatment including subgingival scaling or root planing within the previous 6 months. Before this pilot clinical study, approval was obtained from the Ethics Committee of Stomatological Hospital, College of Medicine, and Xi’an Jiaotong University (No.2016038).

### DPSCs-IPs isolation and cultivation

Inflamed pulps from two patients were extirpated and placed in D-Hank’s solution. A routine RCT was performed. Inflamed pulps were then quickly placed in culture medium for cell isolation. Each sample was first minced and then digested for 1 hour in a solution of collagenase type I and dispase II (3:4) at 37 °C. Cells were then incubated in Dulbecco’s modified Eagle media/Nutrient Mixture F-12 (DMEM/F12 1:1) culture medium with 10 % fetal bovine serum, 2 mmol/L glutamine, 100 μmol/L l-ascorbic acid-2-phosphate, and antibiotics at 37 °C. The colony formation unit-fibroblasts (CFU-Fs) were observed 5 days later.

### Cell Counting Kit-8 assay

The Cell Counting Kit-8 (CCK-8) assay was utilized to detect the viability of DPSCs-IPs, 10^3^ cells/ml were seeded in 96-well plates, and the absorbance at 450 nm was detected at 1–6 days after seeding.

### Osteogenic differentiation

Passage 3 (P3) of DPSCs-IPs was seeded into 12-well plates at a density of 1 × 10^5^ per well and allowed to attach overnight. Next day, the medium was changed for osteogenic differentiation induction medium and then changed every 3 days. Twenty-one days later, cells were stained with Alizarin red.

### Flow cytometry

P3 of DPSCs-IPs were harvested with 0.25 % trypsin, and were incubated for 30 min at 4 °C with primary antibodies. The monoclonal primary antibodies (1:500) were mouse monoclonal anti-human CD44, CD90, CD105, CD117, CD45, CD34, and CD271. Expression profiles were analyzed by flow cytometry (Caliber; BD Biosciences) and the mean fluorescence intensity calculated by flowjo 7.6.3.

### Preparation and evaluation of the DPSCs-IPs/β-TCP complex by scanning electron microscope

Scaffold β-tricalcium phosphate (β-TCP) was placed into dishes when DPSCs-IPs at P3 were at a confluence of 80 %. The medium was generally changed every 3 days. Two weeks later, the complex samples were scraped for scanning electron microscope analysis. They were first put into 2.5 % glutaraldehyde for 2 hours, and then washed with PBS and further fixed with 1 % osmium tetroxide followed by dehydration with ethanol. After displacement, desiccation, and metal spraying, the samples were ready to observe.

### Transplantation of autologous DPSCs-IPs/β-TCP into patients

Patients should undergo initial periodontal therapy before the DPSCs-IPs*/*β-TCP treatment. During transplantation surgery, infiltration anesthesia was used first, and then inflammatory tissues were removed. DPSCs-IPs/β-TCP was transplanted into the periodontal defect areas and sutured carefully.

### Clinical evaluation

The plaque index (PLI), bleeding index (BI), probing depth (PD), gingival recession (GR), clinical attachment level (AL), and tooth mobility were recorded before surgery and post DPSCs-IPs/β-TCP transplantation from 1 to 9 months. All measurements were done with a periodontal probe by blinded examiners.

### Sample collection

Third molars, supernumerary teeth, or teeth removed for orthodontic purposes which were extracted atraumatically from patients were used as the source of normal pulp tissues. We collected inflammatory pulp tissues from teeth diagnosed with irreversible pulpitis. Deciduous teeth were gathered as the source of stem cells from human exfoliated deciduous teeth (SHED).

### Cell cycle analysis

P3 of these cells were trypsined and washed with PBS twice, and were then fixed in 70 % ethanol at 4 °C overnight. The next day, they were washed twice with ice-cold PBS, and stained with PI at a concentration of 50 μg/ml. The stained cells were finally analyzed by flow cytometry.

### In-vitro differentiation and qRT-PCR

P3 of these cells were seeded into six-well plates, and the medium was changed for induction medium for osteogenic differentiation, adipogenic differentiation, and chondrogenic differentiation when the cell confluence reached 90 %. Twenty-one days later, cells were stained with Alizarin red, oil-red O, and toluidine blue to visualize the effect.

Expression levels of ALP, OCN, LPL, PPAR-γ2, COL2A1, and ACAN mRNA were tested after in-vitro differentiation. Primer sequences are presented in Table 1. The protocols used for RNA extraction were similar to those reported previously [12]. Reverse transcription PCR (RT-PCR) was done using a PCR kit (Takara). The quantification process was performed using the SYBR green reagent.

**Table 1:** Primer sequences

	Gene	Gene bank accession number	Primer sequence (5′–3′)	Product (base pair)
Reference	*GAPDH*	NM_002046.3	Forward: GCACCGTCAAGGCTGAGAAC	138
			Reverse: TGGTGAAGACGCCAGTGGA	
Osteogenesis	*ALP*	NM_000478.4	Forward: CATGCTGAGTGACACAGACAAGAA	141
			Reverse: ACAGCAGACTGCGCCTGGTA	
	*OCN*	NM_199173.4	Forward: ATGAGAGCCCTCAGACTCCTC	297
			Reverse: CGGGCCGTAGAAGCGCCGATA	
Adipogenesis	*LPL*	NM_000237.2	Forward: GTCACGGGCTCAGGAGCATTA	144
			Reverse: GCTCCAAGGCTGTATCCCAAGA	
	*PPARγ-2*	NM_015869.4	Forward: TGGAATTAGATGACAGCGACTTGG	182
			Reverse: CTGGAGCAGCTTGGCAAACA	
Chondrogenesis	*ACAN*	NM_001135.3	Forward: ACGAAGACGGCTTCCACCAG	134
			Reverse: TCGGATGCCATACGTCCTCA	
	*COL2A1*	NM_001844.4	Forward: CCAGTTGGGAGTAATGCAAGGA	123
			Reverse: ACACCAGGTTCACCAGGTTCA	

### Statistical analysis

Student’s *t* test and ANOVA test was used. *P* < 0.05 was considered a significant difference.

## Results

### Biological characteristics of DPSCs-IPs in Patient No. 1

**Fig. 1:**
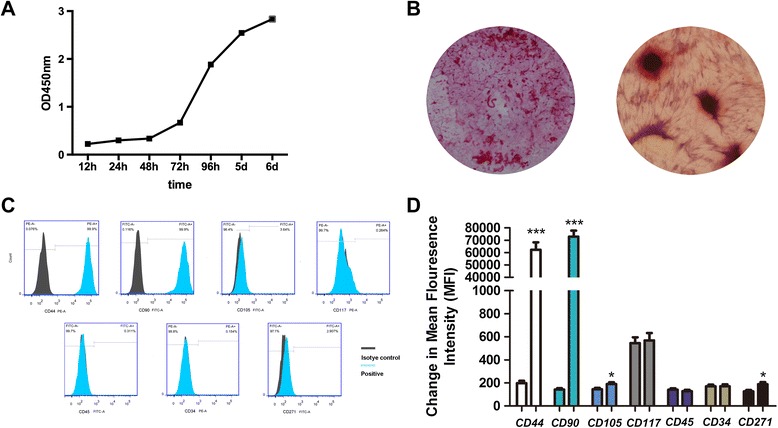
Biological characteristics of DPSCs-IPs in Patient No. 1. **a** CCK-8 assay was utilized to detect the viability of DPSCs-IPs. At days 1–2, DPSCs-IPs stayed in the lag phase, but they showed an elevated proliferation from days 3 to 6. **b** DPSCs-IPs were cultured with osteogenic differentiation medium for 21 days. Alizarin red staining showed mineralized nodules (magnification × 40). **c** Flow cytometry analysis indicated the expression levels of DPSCs-IPs on hematopoietic markers CD34, CD45, and CD117 as well as the mesenchymal stem cell markers CD44, CD90, CD105, and CD271. **d** Mean fluorescence intensity was calculated. (*P < 0.05; ***P < 0.001). Experiments were repeated three times

We objectively evaluated the biological characteristics of DPSCs-IPs in Patient No. 1. Cell growth was observed at the very beginning, and in the first 2 days DPSCs-IPs stayed in the lag phase, while they showed an accelerated proliferation rate from day 3 to day 6 (Fig. 1a). Twenty-one days after osteogenic induction, mineralized nodules were observed by Alizarin red staining (Fig. 1b). Surface molecule expression of DPSCs-IPs is shown in Fig. 1c, d, and hematopoietic markers CD34, CD45, and CD117 together with mesenchymal stem cell markers CD44, CD90, CD105, and CD271 were used to investigate the stem cell properties of DPSCs-IPs.

### DPSCs-IPs/ β-TCP transplantation in Patient No. 1

**Fig. 2:**
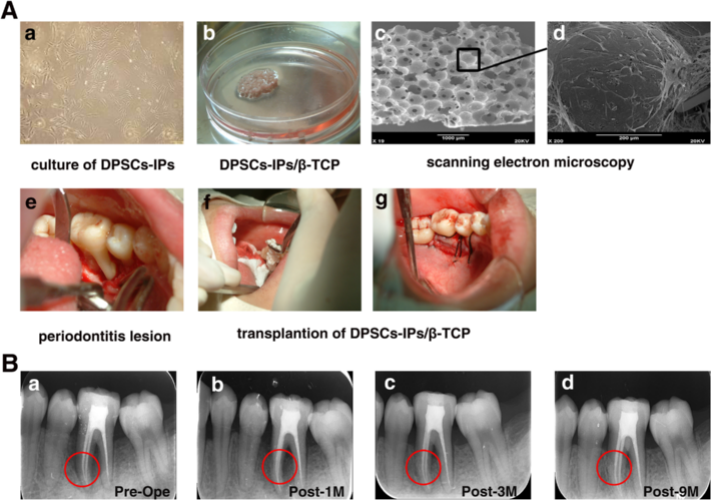
DPSCs-IPs/β-TCP transplantation and therapeutic effect of Patient No. 1. **a** Procedures for DPSCs-IPs/β-TCP transplantation. (*a*) Third passage of DPSCs-IPs from Patient No. 1. (*b*) Generation of DPSCs-IPs/β-TCP complex. DPSCs-IPs were cultured in 100-mm culture dishes with 40 mg β-TCP particles. (*c*, *d*) Scanning electron microscopy of DPSCs-IPs/β-TCP complex. (*e*) Lingual view of periodontitis lesion. (*f*, *g*) Transplantation of the DPSCs-IPs/β-TCP complex generated from Patient No. 1 into the periodontal lesion. **b** Therapeutic effect of DPSCs-IPs/β-TCP in Patient No. 1. (*a*) Bone defeats before the operation (*red circle* in *Pre-Op*). (*b*) Therapeutic effect 1 month after the operation (*red circle* in *Post-1 M*). (*c*) Therapeutic effect 3 months after the transplantation (*red circle* in *Post-3 M*). (*d*) Therapeutic effect 9 months after the operation (*red circle* in *Post-9 M*) by X-ray analysis. *DPSCs-IPs* dental pulp stem cells isolated from inflammatory dental pulp tissues, *β-TCP* β-tricalcium phosphate

Figure 2A clearly shows the protocol of a procedure for using DPSCs-IPs from patients to treat periodontal bone defeats. DPSCs-IPs from Patient No. 1 were cultured to the third passage (Fig. 2Aa). All procedures were done with the patient’s agreement and her knowledge. To prepare the DPSCs-IPs/β-TCP complex, DPSCs-IPs were cultured in a 100-mm dish for 3 days, and 40 mg β-TCP particles were added to the dishes; 2 weeks later, the complex samples were ready (Fig. 2Ab). We used scanning electron microscopy to detect the DPSCs-IPs/β-TCP complex (Fig. 2Ac, Ad). After removing infectious periodontal tissues, the DPSCs-IPs complex was applied to the periodontal bone defective areas (Fig. 2Ae–g).

After transplanting in-vitro expanded DPSCs-IPs/β-TCP into the intrabone defects of deep periodontal pockets in Patient No. 1 following the standard surgical debridement, the patient was monitored carefully and followed up at 1, 3, and 9 months. Routine clinical evaluations including PD, AL, and GR were examined and X-ray scans were taken at 1, 3, and 9 months after surgery (Table 2 and Fig. 2B).

**Table 2:** Clinical characteristics of Patient No. 1

Clinical index	Before treatment	1 month after treatment	3 months after treatment	9 months after treatment
Dental plaque	3	2	1	1
Sulcus bleeding index	3	2	1	1
Gingival recession (mm)	3	2.5	2.5	2.5
Probing depth (mm)	6	4	3	3
Furcation lesion (degree)	III	II	II	II
Mobility (degree)	III	II	I	I

### DPSCs-IPs/β-TCP transplantation in Patient No. 2

**Fig. 3:**
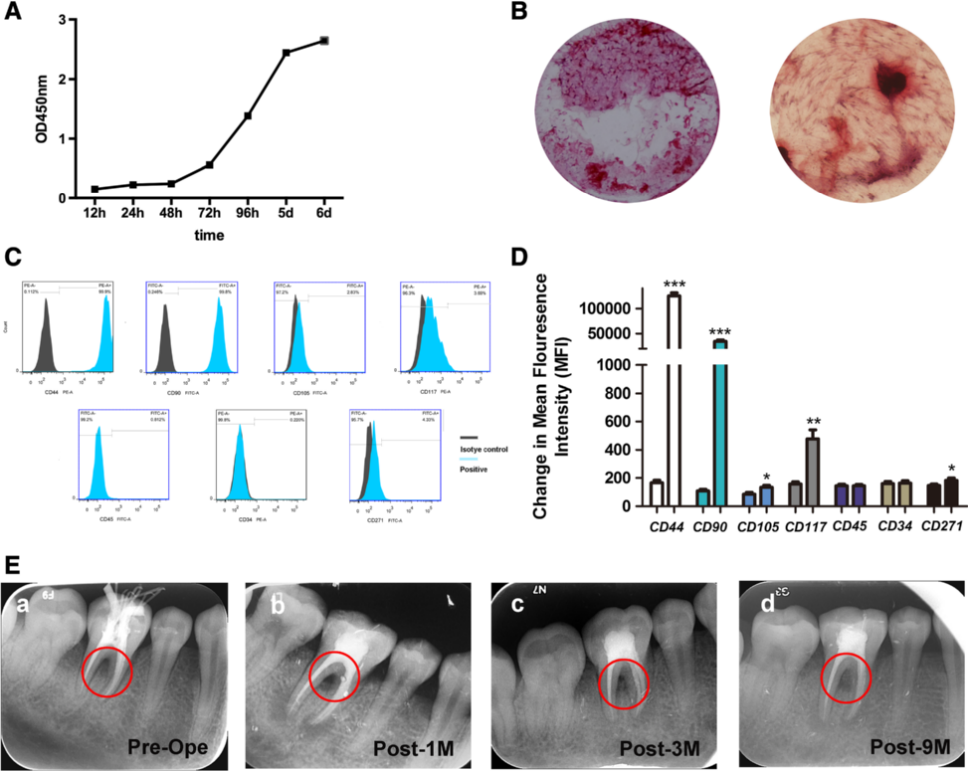
DPSCs-IPs and the therapeutic effect of Patient No. 2. **a** Viability of DPSCs-IPs in Patient No. 2. The proliferation status of DPSCs-IPs in Patient No. 2 was similar to that in Patient No. 1. **b** Mineralized nodule formation can be observed 21 days after osteogenic induction (magnification × 40). **c** DPSCs-IPs in Patient No. 2 were negative to hematopoietic markers, but positive to mesenchymal stem cell markers. **d** Mean fluorescence intensity was also calculated (*P < 0.05; ***P < 0.001). Experiments were repeated three times. **e** Therapeutic effect of DPSCs-IPs/β-TCP in Patient No. 2. (*a*) Bone defeats before the operation (*red circle* in *Pre-Op*). (*b*) Therapeutic effect 1 month after the operation (*red circle* in *Post-1 M*). (*c*) Therapeutic effect 3 months after the transplantation (*red circle* in *Post-3 M*). (*d*) Therapeutic effect 9 months after the operation (*red circle* in *Post-9 M*) by X-ray analysis

The biological characteristics of DPSCs-IPs in Patient No. 2 were also evaluated (Fig. 3A–D), and the DPSCs-IPs/β-TCP complex was prepared as described previously. X-ray scans were taken at 1, 3, and 9 months after surgery (Fig. 3E). The proliferation status of DPSCs-IPs in Patient No. 2 was similar to that in Patient No. 1. Mineralized nodule formation can be observed 21 days after induction and cells were negative to hematopoietic markers, but positive to mesenchymal stem cell markers.

More importantly, DPSCs-IPs also showed an effective therapeutic effect in Patient No. 2 (Table 3 and Fig. 3E).

**Table 3:** Clinical characteristics of Patient No. 2

Clinical index	Before treatment	1 month after treatment	3 months after treatment	9 months after treatment
Dental plaque	2	1	1	1
Sulcus bleeding index	3	2	1	1
Gingival recession (mm)	1	1	1	1
Probing depth (mm)	5	4	3	3
Furcation lesion (degree)	III	II	I	I
Mobility (degree)	II	II	I	I

### Phenotypes of three kinds of DPSCs

**Fig. 4:**
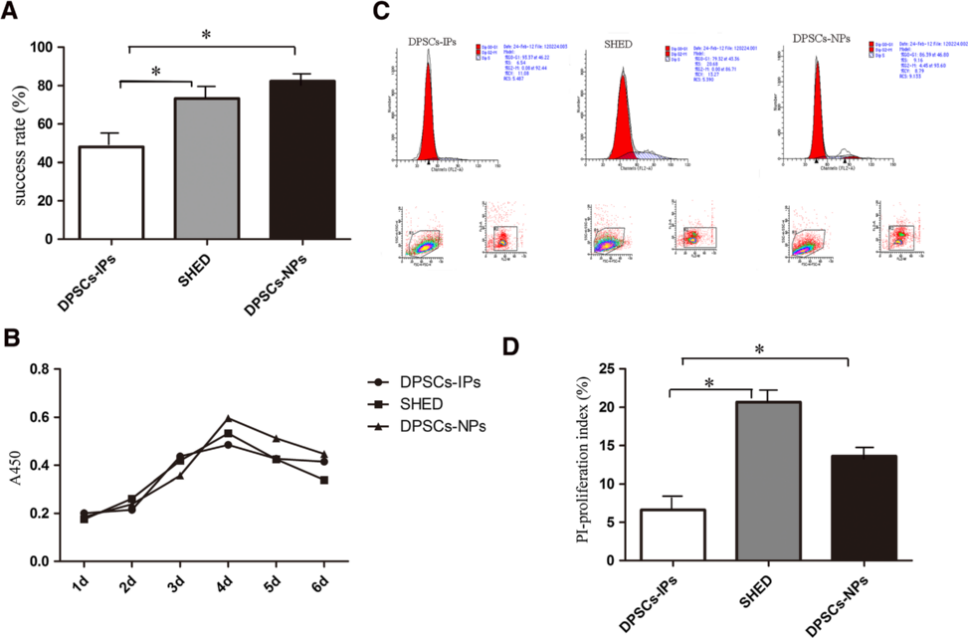
Success rate of primary cell culture and growth state of DPSCs-IPs compared with DPSCs-NPs and SHED. Primary cell culture success rate of three kinds of dental pulp-derived stem cells. **a** Cell growth curves of three kinds of dental pulp-derived stem cells. **b** Cell cycle determination of three kinds of dental pulp-derived stem cells. **c** Cell proliferation rate of three kinds of dental pulp-derived stem cells. **d** Cell proliferation index of three kinds of dental pulp-derived stem cells. *P < 0.05. *DPSCs-IPs* dental pulp stem cells isolated from inflammatory dental pulp tissues, *DPSCs-NPs* dental pulp stem cells from normal dental pulp tissues, *SHED* stem cells from human exfoliated deciduous teeth

As observed previously, DPSCs-IPs have the ability to restore periodontal bone defeats in two patients; it is interesting to discuss the biological phenotype of DPSCs-IPs compared with two other kinds of DPSCs. We therefore used normal human dental pulp stem cells (DPSCs-NPs) and deciduous dental pulp stem cells (SHED) to evaluate the phenotype of DPSCs-IPs (details of sample collection are presented in Table 4). The growth state was evaluated by the success rate of primary culture, the cell proliferation index PI = (G2/M + S) / (G0/G1 + S + G2/M) × 100 %, and the cell growth curve. The results showed that in DPSCs-IPs, compared with the two other kinds of normal cells, the primary culture success rate decreased (Fig. 4a), cell growth was slightly restricted (Fig. 4b), and the cell proliferation index was significantly decreased (Fig. 4c, d). In summary, although the growth state of DPSCs-IPs is slightly influenced, these cells can still be successfully cultured and amplified.

**Table 4:** Statistical table of dental pulp tissues

Category	Total number	Success number	Success rate	Average age	Position	Primary clone number (mean ± SD)
Inflammatory dental pulps	25	12	48.00 %	26	Anterior teeth (68.00 %)	33.08 ± 4.963
					Molar teeth (32.00 %)	
Deciduous dental pulps	30	22	73.33 %	7	Lower anterior teeth	65.41 ± 6.404
Normal dental pulps	34	28	82.35 %	20	Orthodontic teeth (63.33 %)	68.68 ± 6.043
					Wisdom teeth (36.67 %)

### Assessment of the ability of multidirection differentiation of DPSCs-IPs

**Fig. 5:**
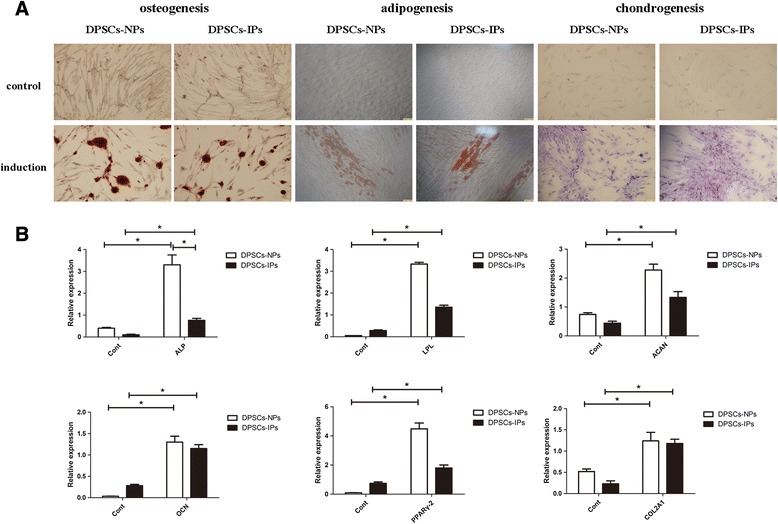
Assessment of multidirection differentiation abilities of DPSCs-IPs. **a** Induced osteogenesis, adipogenesis, and chondrogenesis were shown 21 days after induction. *Scale bars* = 100 mm. **b** Expression levels of ALP, OCN, LPL, PPAR-γ2, COL2A1, and ACAN mRNA of DPSCs-IPs after in-vitro differentiation. *P < 0.05. *DPSCs-IPs* dental pulp stem cells isolated from inflammatory dental pulp tissues, *DPSCs-NPs* dental pulp stem cells from normal dental pulp tissues

Next, we further tested the ability of multidirection differentiation of DPSCs-IPs and related gene expression. DPSCs-IPs were positively stained in osteogenesis, adipogenesis, and chondrogenesis (Fig. 5a). Gene markers were examined by qRT-PCR at 21 days after in-vitro differentiation (Fig. 5b). We found that the ability of osteogenic differentiation of DPSCs-IPs slightly decreased, while the adipogenic and chondrogenic differentiation ability showed no obvious difference compared with DPSCs-NPs. These results suggest that although osteogenic ability is affected to some extent, DPSCs-IPs still could be a choice for the replacement of DPSCs-NPs in clinical practice.

## Discussion

Previous studies have shown that although they lose some of the properties of stem cells, DPSCs-IPs retain the potential for tissue regeneration [9, 10, 13].

In the present study, DPSCs-IPs were transplanted into the patients’ periodontal bone defects for the first time and the effective repairing effect was observed.

To date, only a few in-vivo studies in patients have been reported in oral tissue regeneration instead of only discussing the biological characteristics of the stem cells. There are many reasons for this lack of research, but what is at least certain is that normal dental stem cells need to be obtained from normal tissues, a process which itself would be new damage for patients. In this case, patients often refused the treatment. However, DPSCs-IPs themselves are derived from the inflammatory dental pulp tissues that are always taken as medical waste, so it is acceptable for patients to agree with this kind of treatment.

This study was the first to complete bone regeneration by autologous transplantation of DPSCs-IPs in patients. We objectively evaluated the characteristics of DPSCs-IPs in each patient first. The study found that inflammatory dental pulp tissues in both patients to a certain extent retain the properties of DPSCs: they can differentiate into osteogenic cells, and they express certain surface markers of mesenchymal stem cells. The expression levels in CD44 and CD90 are highly positive, and the levels in CD34 and CD45 are negative, which is in line with the characterization of mesenchymal stem cells. But the levels in CD105 and CD271 are weak, which slightly differs from previous reports [14–16]. However, the underlying reason remains unclear. The property of stem cell markers in different species or organs indeed differs in some cases [9]. Using the expression levels in CD44, CD90, CD34, and CD45, however, the stem cell properties of DPSCs-IPs can be determined. The following discusses the therapeutic effect of DPSCs-IPs from many aspects. We have provided evidence here that the dental clinical condition was improved obviously 9 months after transplantation of the DPSCs-IPs/β-TCP complex. As observed in the clinic, the color of the gum is pink, and its quality is tough and elastic. Although there is only an inconspicuous improvement in GR, the PD was evidently shallow, the gingival BI decreased from 3 to 1, clinical hemorrhage disappeared, the root bifurcation lesions reduced to degree II–I compared with degree III before treatment, and the treatment effect was obvious from the current clinical symptoms. Generally speaking, the DPSCs-IPs/β-TCP autograft dramatically improved the clinical symptoms of periodontitis. Our results provide evidence that DPSCs-IPs/β-TCP compounds may have a certain repair effect on periodontal hard tissue defects caused by periodontitis and may be a new source for oral tissue regeneration to provide a potential way of being used in future clinical applications.

β-TCP has been used in tissue engineering for a long time, it has excellent bone conductibility, biological activity, and mechanical performance, and it has certain ability to repair bone defeats combined with stem cells [17–20]. In our study, DPSCs-IPs can be well engrafted into β-TCP, and no side effect or uncomfortable feelings appeared in patients after the transplantation. Therefore it is suggested that β-TCP can be used as a good carrier for tissue repair in the future.

In the view of safety in the process of transplantation, no patients showed any systemic disorders related to the transplantations or adverse reactions during the process, so the procedures used in this study can benefit DPSCs-IPs clinical studies in the future.

By further comparing the biological characteristics of DPSCs-IPs with two kinds of normal DPSCs, we found that although DPSCs-IPs can be isolated from inflammatory dental tissues, their growth state is inhibited to some extent due to the inflammatory source, which is in line with previous reports [21–23]. However, despite the decreased osteogenic ability compared with normal DPSCs, the ability to differentiate into osteogenic, adipogenic, and chondrogenic cells proved the characteristics of stem cells and suggests the potential of mesenchymal stem cells to repair defeats.

Despite these promising results, the flaws of this study reside mainly in the definite mechanism of DPSCs-IPs-mediated regeneration and the small number of patients enrolled. Future studies should concentrate on the specific mechanism of DPSCs-IPs-mediated tissue regeneration and include more clinical studies with large numbers of patients.

## Conclusions

In this study, we provide early clinical data as well as experimental evidence to support the efficacy and safety of application of autologous DPSCs-IPs related to human periodontitis treatment of bone defect. We speculate that DPSCs-IPs can be a suitable cell source and can be isolated from dental pulps exhibiting inflammation, and we also speculate that DPSCs-IPs will perform excellent effects in the treatment of periodontal regeneration. We hope to design a clinical trial in the future with a large number of patients to provide further information about DPSCs-IPs treatment.

## Abbreviations

AL: Clinical attachment level
BI: Bleeding index
CCK-8: Cell Counting Kit-8
CFU-F: Colony formation unit-fibroblast
DMEM/F12: Dulbecco’s modified Eagle media/Nutrient Mixture F-12
DPSC: Dental pulp stem cell
DPSCs-IPs: Dental pulp stem cells isolated from inflammatory dental pulp tissues
DPSCs-NPs: dental pulp stem cells from normal dental pulp tissues
GR: Gingival recession
PD: Probing depth
PLI: Plaque index
RT-PCR: Reverse transcription PCR
SHED: Stem cells from human exfoliated deciduous teeth
β-TCP: β-tricalcium phosphate
